# Exploring variation in the six-month review for stroke survivors: a national survey of current practice in England

**DOI:** 10.1186/s12913-025-12323-6

**Published:** 2025-01-28

**Authors:** Rich Holmes, Suzanne Ackerley, Rebecca J Fisher, Louise A Connell

**Affiliations:** 1 Physiotherapy Department, St Richard’s Hospital, University Hospitals Sussex NHS Foundation Trust, Chichester, West Sussex PO19 6SE UK; 2 Lancaster University, Lancaster, UK; 3 University of Central Lancashire, Preston, UK; 4 Stroke Programme, King’s College London, London, UK; 5 East Lancashire Hospitals NHS Trust, Burnley, UK

**Keywords:** Six-month review, Stroke rehabilitation, Life after stroke, Implementation of healthcare policy, Follow-up care, Health inequality

## Abstract

**Background:**

The Six-Month Review (6MR) was introduced in the United Kingdom to provide a holistic, systematic review of the ongoing needs faced by stroke survivors. However, a theoretical underpinning regarding how it should work is lacking, potentially leading to wide variation in service provision. This study aimed to understand the current degree of variation in 6MR delivery across England and explore the potential driving factors.

**Methods:**

A cross-sectional study was conducted via an online survey distributed to 6MR services within England. Data were collected over 12 weeks in 2023. Descriptive statistics were used to demonstrate the degree of variation in service delivery, and associations were explored between features of the 6MR service and contextual factors.

**Results:**

Ninety-two responses were received, representing approximately 53% of 6MR services in England. Wide variation was observed in relation to service structure, content and processes, and in how outcomes, experience and effectiveness are measured. A number of significant associations were observed between features of the 6MR and contextual factors, most commonly, in relation to the provider organisation.

**Conclusions:**

This study highlights the degree of variation in 6MR delivery across England. The provider organisation may be a driving factor for this variation that warrants further investigation. Future research should focus on understanding how, and under what circumstances, the 6MR works so that its effectiveness can be evaluated and best practice established.

**Supplementary Information:**

The online version contains supplementary material available at 10.1186/s12913-025-12323-6.

## Background

By 2025, in the United Kingdom, it is projected that there will be 1.4 million stroke survivors living with the effects of their stroke [1]. This prevalence is anticipated to increase further to 2.1 million with societal costs reaching £75 billion by 2035 [1]. The long-term problems and unmet needs faced by stroke survivors are extensive and multifaceted, encompassing physical, cognitive, psycho-emotional, and social aspects [2, 3]. The situation is further complicated by the fact that certain factors, such as ethnicity, level of disability and socioeconomic status, impact the nature and number of perceived needs of stroke survivors [4]. It is, therefore, vital that stroke services are able to effectively address the disabling effects of stroke, as well as secondary complications, so that the burden to the individual and society is limited.

Addressing heterogenous problems requires a holistic, personalised and systematic approach to identify unmet needs followed by actions taken to ameliorate them. In view of this, the National Stroke Strategy [5] first recommended that all stroke survivors should be offered a review of their health and care status six months after leaving hospital, since termed the Six-Month Review for Stroke Survivors (6MR). Subsequent national guidelines [6–8] further reiterated this intention with the most recent highlighting the need for personalisation and flexibility within the review process. However, after 17 years, and despite financial incentives [9], delivery of the 6MR remains poor with only 37% of eligible stroke survivors receiving the review [10].

National stroke guidelines [6–8] are limited to statements that the 6MR *should* be delivered rather than providing details of *how* it should be delivered. Recommendations for the 6MR were based mostly on expert consensus in lieu of an evidence base specifically defining its purpose and remit or demonstrating its effectiveness in achieving anticipated outcomes. Randomised controlled trials investigating 6MR effectiveness found no evidence of clinical benefit in relation to activities of daily living and well-being [11, 12]. However, such evaluations are constrained by the lack of a programme theory for the 6MR and because anticipated outcomes have not been clearly defined.

The 6MR can be described as a complex intervention as defined by the Medical Research Council [13] given the range of behaviours targeted, the heterogeneity of the target group, the range of skills required from those delivering the intervention to ensure an individualised approach, and the high potential for the intervention to interact with the context into which it is placed. As such, it would benefit from a theoretical understanding regarding how, and under what circumstances, it should work [14]. Programme theory of this nature would provide the much-needed clarity required for wide scale implementation by articulating the core components of the intervention and establishing the elements that are adaptable to local context [15]. However, such theoretical underpinning is currently lacking within the literature and the intervention remains poorly defined, exacerbating the potential for varied implementation.

An audit of 6MR services within England was undertaken in 2014 [16]. This project aimed to map the provision of services at that time and to understand what structures and processes had been implemented. The findings demonstrated a high degree of variation in provision, with numerous models of delivery adopted, suggesting a lack of clarity around the ideal structure and content of the 6MR. A decade later, further mapping is warranted to recognise if subsequent clinical guidelines and the establishment of 6MR services over time have altered the degree of variation in service delivery.

With this in mind, this study aimed to understand what variation currently exists in the delivery of 6MR services across England, and to explore what factors might be driving this variation. This is a necessary first step towards being able to determine the core components of the 6MR so that future work can begin to build programme theory for subsequent evaluations.

## Methods

### Study design

A cross-sectional study was undertaken with data collected from 6MR services in England via an online survey. Data was collected for a 12-week period in 2023. The survey comprises the initial stage of an explanatory-sequential mixed methods study (BE MoRe: Exploring the Benefits and Expectations of the 6-Month Review for Stroke Survivors). Ethical approval for this study was obtained from the HEALTH Ethics Review Panel at the University of Central Lancashire (Reference number: HEALTH 0401 WP1).

### Survey development

A survey was developed using the online tool, Qualtrics® (Qualtrics, Provo, UT). The survey was structured in three sections: (1) *Service structure, content and processes*, (2) *Populations served*, and (3) *Purpose and outcomes*. A previous national audit of services [16] was used as a basis for survey content with elements updated to reflect advancements in technology now used in common practice (i.e. virtual assessments). Three domains (‘Innovation’, ‘Outer Setting’ and ‘Individuals – Subdomain: Characteristics’) of the updated Consolidated Framework for Implementation Research (CFIR) [17] were used as a conceptual framework to develop questions. Additional questions, specifically regarding content and purpose of the 6MR, were informed by reviewing the evidence base (including grey literature such as service specifications and commissioning documents [9, 18, 19]) and via feedback from clinicians and service users in the refinement stage. During this stage, the online survey was piloted on a group of 6MR providers (NHS *n* = 2, charitable organisation *n* = 3), and clinical academics with experience of survey design (*n* = 2). Minor changes were made to the structure of questions based on feedback. A second round of piloting was undertaken (*n* = 3) to check clarity, usability and time taken to complete. The final survey utilised a mix of rating scales, Likert scales, multiple-choice, and open-ended questions (see Additional file 1).

### Participants

The target population was service leads of 6MR services within England. The most recent Sentinel Stroke National Audit Programme (SSNAP) Post-Acute Organisational Audit [20] reports 174 6MR providers within England. Informed consent to participate was obtained from all participants. Respondents were excluded from the survey if their service did not provide 6MRs or if they were not someone who had responsibility for delivering the 6MR. Respondents were asked to provide the name of their service to avoid duplicate responses from each service but, to ensure anonymity, this was stored separately to the rest of the dataset. Given the specific target population and anticipated range of service providers, a multi-faceted recruitment approach was undertaken to ensure an adequate response rate. A link to the online survey was placed on Twitter and on an online networking platform. The link was also included in national newsletters with a focus on stroke rehabilitation. Alongside this link was a request to share the survey with other services to increase recruitment via snowball sampling. Further recruitment was supported by regional leaders in stroke rehabilitation who were able to use a more targeted approach to 6MR providers within their regions.

### Data analysis

Data was collected using Qualtrics® and imported to IBM SPSS Statistics (Version 28) for analysis.

Descriptive statistics were used to display the frequency that different features (i.e. the method of delivery, where the 6MR took place, etc.) were present within the 6MR services surveyed. Frequency tables were also used to demonstrate the occurrence rate of contextual factors (including both service characteristics and sociodemographic factors). To begin to understand the potential drivers behind variation, associations were explored between features of the 6MR service and contextual factors using Fisher’s Exact test. The service characteristics explored were the age of the service (≤10 years, >10 years) and the provider organisations of the service (Acute NHS Trusts, Community NHS Trusts, Charitable Organisations and Other). The sociodemographic factors explored were the degree of rurality (rural, semi-rural and city), the level of deprivation (high, moderate and low), and the age distribution of the local caseload (older population, younger population; dichotomised to ‘younger population’ if 40% or more of the typical caseload was under 65 years of age). To maintain anonymity, respondents were asked to subjectively identify the sociodemographic factor that best characterised the majority of their local population rather than being linked to objective data.

The time taken to complete the review process was summarised using the median and interquartile range. Differences between contextual factors in relation to the time taken were explored through the Kruskal-Wallis H test and the Mann-Whitney U test.

All comparisons were performed using two-tailed tests with a p-value <.05 considered statistically significant.

## Results

### Responses

**Fig. 1:**
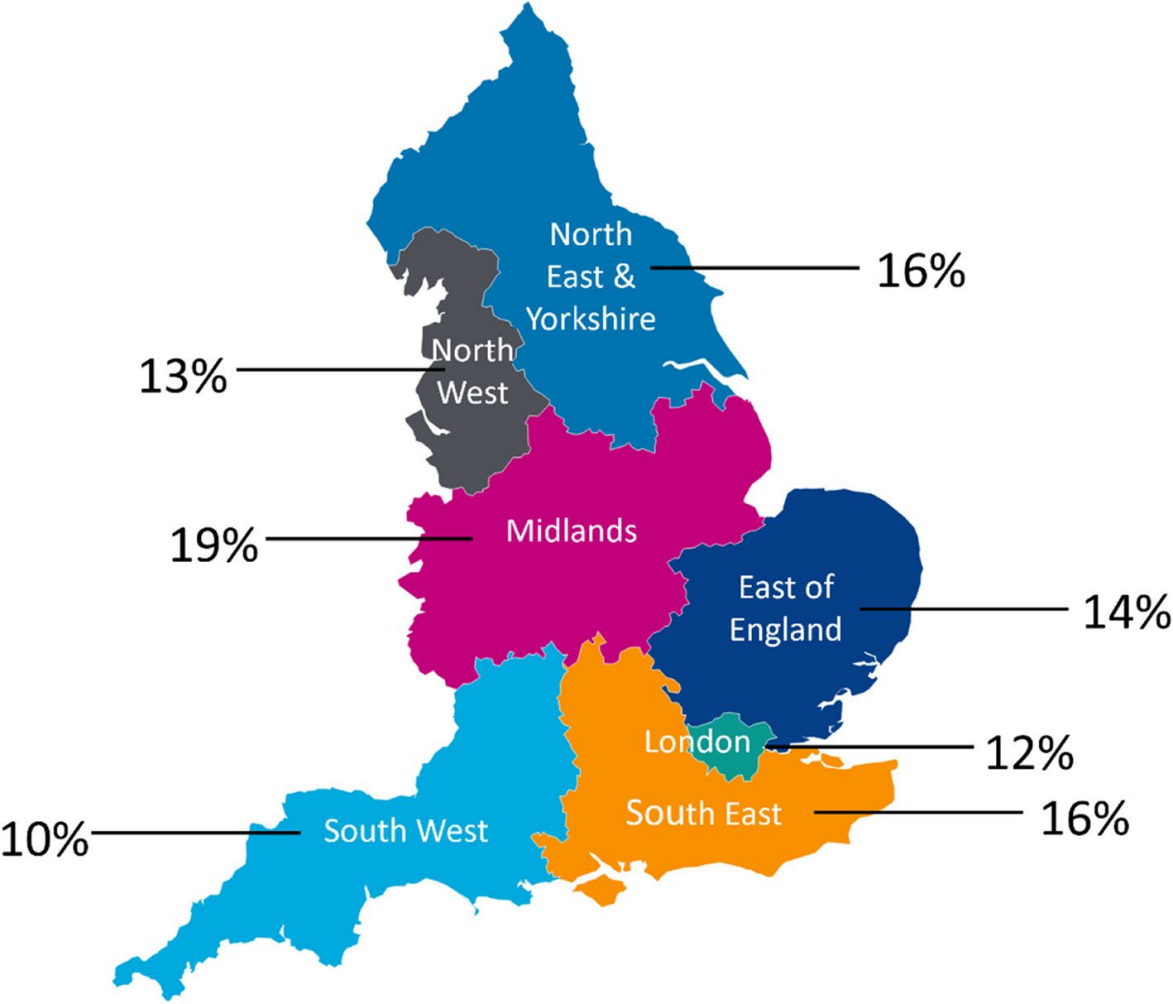
Responses per region (*n* = 92*)*

A total of 92 services submitted surveys met the inclusion criteria giving an estimated response rate of 53%. Responses were received from all 20 Integrated Stroke Delivery Networks within England. The spread of responses across regions can be seen in Fig. 1.

### Service characteristics

Most services had been established for over 10 years (55%) and services were most often provided by Community NHS Trusts (41%). Further frequencies for the age of the service and the provider organisation can be seen in Table 1 alongside the professionals involved in the services. A third (34%) of services were multidisciplinary whereby they reported multiple professions were involved in delivering the review. Further information regarding the range of professions within each provider organisation can be seen in an additional file (see Additional file 2).

**Table 1:** Characteristics of 6MR services

		% *(n), n=92*
Age of Service (years)	0–3	12 *(11)*
	4–5	8 *(7)*
	6–10	25 *(23)*
	Over 10	55 *(51)*
Service Provider	Community NHS Trust	41 *(38)*
	Acute NHS Trust	21 *(19)*
	Charitable Organisation	29 *(27)*
	Community Interest Company	5 *(5)*
	Private Company	1 *(1)*
	Other	2 *(2)*
Professionals Involved	Nurse	51 *(47)*
	Charitable Sector Employee	29 *(27)*
	Allied Health Professional	25 *(23)*
	Support Worker / Assistant	22 *(20)*
	Consultant	4 *(4)*
	Psychologist	1 *(1)*

### Service structure, content and processes

An overview of the frequency of the features of 6MR service delivery can be seen in Table 2. The majority of services offered face-to-face (93%) and telephone (89%) appointments to carry out the 6MR. Virtual appointments were offered by 37% of services. The majority of services offered a degree of flexibility within their delivery methods, with over 80% offering at least two different approaches. The most common location to carry out the review was within the stroke survivor’s own home with 89% of services offering this. In contrast, only 40% delivered the 6MR within residential or nursing homes.

**Table 2:** Frequency of features of 6MR service delivery

		% *(n), n=92*
Method of Delivery	Face-to-face	93 *(86)*
	Telephone	89 *(82)*
	Virtual	37 *(34)*
	Post	4 *(4)*
Number of Different Delivery Methods	1	17 *(16)*
	2	42 *(39)*
	3	39 (*36)*
	4	1 *(1)*
Locations Offered	Home	89 *(82)*
	Residential / Nursing Home	40 *(37)*
	Clinic	34 *(31)*
	Community Centre	3 *(3)*
	GP Surgery	1 *(1)*
	Not applicable (no reviews offered face to face)	4 *(4)*
Data Collection Tool	GM-SAT	59 *(54)*
	‘In-house’ / Self-Devised	38 *(35)*
	PSC	3 *(3)*
	LUNS	1 *(1)*
	Other	24 *(22)*
	No tool used	2 *(2)*

*GM-SAT* Greater Manchester Stroke Assessment Tool, *PSC* Post-stroke Checklist, *LUNS* Longer-term Unmet Needs after Stroke

The checklist or data collection tool most commonly used to support the review process was the Greater Manchester Stroke Assessment Tool (GM-SAT) with 59% of services utilising it. Of the responses that reported ‘Other’ data collection tools were used, five reported using adapted versions of the GM-SAT and four reported using the Wessex Stroke Review Tool. Just over a quarter (26%) of services used more than one data collection tool, however data was not collected during the survey to understand why this was felt necessary.

The median direct time spent with the stroke survivor to complete the 6MR was 60 minutes (IQR: 45–60 minutes) and the median indirect time for associated paperwork and referrals was 45 minutes (IQR: 25–75 minutes).

The percentage of 6MR services that review the various needs and issues is reported in an additional file (see Additional file 3). The majority of needs investigated by the survey were reported to be reviewed by over 85% of services. However, spasticity was reported to have been reviewed by only 66% of 6MR services. ‘Other’ needs that were reported to be reviewed by the 6MR included blood pressure checks, altered sensation, seating needs, oedema management and nutrition.

Four of the 92 surveyed services (three acute providers and one community provider) reported that they did not make any direct onward referrals as a result of the 6MR. Most commonly, 6MR services were able to refer directly to Speech and Language Therapy (84%), Community Rehabilitation (82%), Incontinence Services (79%), and Carer’s Support (79%). Fewer 6MR services reported referrals directly to Inpatient Rehabilitation (15%), Audiology (23%), and Pain Clinic (23%). More detail regarding onward referrals is reported as an additional file (see Additional file 4). Respondents were given the option to describe ‘Other’ referrals not previously mentioned in the survey. Responses to this included sleep clinics, wellbeing services, various group activities, financial support, charitable organisations, podiatry, and tissue viability nurses. No data was collected during the survey that assessed the appropriateness of onward referrals.

### Populations served

The frequency of respondents who reported that their 6MR service covered these populations (described as sociodemographic factors) can be seen in Table 3.

**Table 3:** Socio-demographic factors

		% *(n), n=92*
Rurality	Rural	10 *(9)*
	Semi-rural	42 *(39)*
	Urban / city	48 *(44)*
Levels of Deprivation	Low	16 *(15)*
	Moderate	61 *(56)*
	High	23 *(21)*
Age Distribution	Younger population	39 *(36)*
	Older population	61 *(56)*

Respondents were asked to report on any groups within their areas that they felt were underserved by current services. The main groups highlighted were those who did not speak English and those from ethnic and cultural backgrounds other than ‘White British’. Other common themes included those with communication or cognitive difficulties, and those who live in care homes. Groups that were mentioned less frequently or by a single respondent included: individuals with difficulty accessing transport, individuals who did not have the support of a next of kin, and people with mental health problems.

Approximately half (49%) of services reported collecting data on stroke survivors who didn’t take up the offer of the 6MR. The most often collected data was a simple count and the reasons for non-uptake. Only four services reported collecting any demographic data to explore any patterns in non-uptake.

### Purpose and outcomes

Respondents were asked to select up to three options of what they felt were the main purposes of the 6MR. The most commonly selected purposes were to ‘Identify unmet needs’ (85%), to ‘Provide information/advice/signposting’ (70%), and ‘Secondary prevention’ (54%). Other options were selected less frequently. These were: to ‘Provide personalised care’ (16%), to ‘Provide emotional support’ (16%), for ‘Data gathering to inform commissioning need’ (15%), for ‘Onward referral to specialist services’ (13%), for ‘Onward referral to rehabilitation’ (9%), to ‘Identify carer needs’ (9%), and to ‘Provide intervention/treatment’ (2%).

In terms of assessing outcomes in the 6MR, 62% of respondents reported using a validated outcome measure. Of those services that reported that they did use a validated outcome measure there was a large variation in outcome measure selection, with 15 different measures reported. The most common outcome measures used were the modified Rankin Scale and the EQ5D-5L, reflecting the requirements of SSNAP data entry requirements. Similarly, 65% of 6MR services measured patient experience or satisfaction levels. Again, there was variation in how this was measured with some services using a simple rating scale and others describing more in-depth questionnaires.

Overall, 38% of services reported that the success of their service had been evaluated in some way. However, of these, the majority were either via SSNAP reports, via informal reports of success, or respondents were not sure how the evaluation had occurred. Only three respondents described any kind of report related to performance and quality of the service.

### Potential drivers of variation

Statistical analysis was conducted to investigate associations between selected contextual factors and the features of the 6MR service. These associations were used to explore which factors might explain the observed variation in practice. Table 4 shows which of these pairings were significantly associated. For example, the statistically significant value between face-to-face delivery and provider organisation demonstrates that the provision of this delivery method was closely associated with who the provider organisation was, whilst other contextual factors had no bearing on whether this method was used or not. As can be seen from Table 4 the majority of significant associations were present in relation to the provider organisation suggesting this factor may warrant further investigation as a potential driver of the observed variation.

**Table 4:** Significant associations between features of the 6MR service and contextual factors

		**Contextual factors**				
		Sociodemographic factors			Service characteristics	
		Rurality	Level of deprivation	Age distribution	Provider organisation	Age of service
**Features of 6MR**						
Method of Delivery	Face-to-face	-	-	-	.022	-
	Telephone	-	-	.044	-	-
	Virtual	-	-	-	<.001	-
Location	Home	-	-	-	<.001	-
	Clinic	-	-	-	<.001	-
Data Collection Tool	GM-SAT	-	-	-	<.001	-
	‘In-house’ forms	-	-	-	<.001	-
Direct Referrals	Carer’s Support	-	-	-	-	.022
	Clinical Psychologist	-	-	-	.010	.035
	Dieticians	-	-	-	.043	-
	Driving Assessment	-	-	-	-	.003
	Social Worker	-	.003	-	.006	-
	Spasticity	-	-	-	.007	-

*GM-SAT* Greater Manchester Stroke Assessment Tool

NB. Only features with at least one statistically significant association have been included. All other features did not show any significant association with any of the contextual factors

The time taken to complete the 6MR was compared between groups within the different contextual factors. No significant differences were observed when exploring sociodemographic factors but there were significant differences noted when exploring service characteristics. The time taken to undertake the 6MR was significantly different between provider organisations (H(5) = 18.896, *p* = .002) as was the indirect time spent completing associated paperwork (H(5) = 22.998, *p* < .001). Post-hoc testing showed that, for both direct and indirect time, acute NHS trusts spent significantly less time than both charitable organisations (*p* < .001) and community NHS trusts (*p* < .003). Comparisons of time taken to complete can be seen in Table 5.

**Table 5:** Frequency of the presence of service features per provider organisation

		Acute NHS trusts (*n=19*)	Community NHS trusts (*n=38*)	Charitable organisations (*n=27*)	Others (*n=8*)
Method of Delivery	Face-to-face	74%	97%	100%	100%
	Telephone	95%	79%	96%	100%
	Virtual	5%	29%	70%	38%
Location	Home	58%	95%	100%	100%
	Clinic	47%	47%	0%	50%
	Residential / Nursing Home	21%	42%	44%	63%
Data Collection Tool	GM-SAT	37%	34%	100%	75%
	‘In-house’ forms	53%	55%	7%	25%
Time to Complete (Minutes) – *Median (IQR)*	Direct	35 (30–60)	60 (45–60)	60 (50–90)	60 (55–67.5)
	Indirect	20 (10–30)	45 (30–60)	60 (45–120)	37.5 (30–75)
Number of Different Delivery Methods – *Median (IQR)*		2 (1–2)	2 (1.75–3)	3 (2–3)	2 (2–3)

*IQR* Inter Quartile Range, *GM-SAT* Greater Manchester Stroke Assessment Tool

The time taken to undertake the review (≤10 years: median = 60 minutes; >10 years: median = 53 minutes) was significantly greater in those services that were ≤10 years (Mann-Whitney *U* = 763.5, *n*_*1*_ = 51, *p* = .021 two-tailed). The indirect time spent on the review was not significantly different for this factor.

As the majority of significant associations were observed in relation to the provider organisations, further data showing the frequencies of service features is provided in Table 5 for comparison. An expanded version of Table 5 (including comparisons of non-significant features) is available in an additional file (see Additional file 2).

## Discussion

The findings demonstrate a large variation in the delivery of the 6MR in terms of structure, content and process. This adoption of a wide range of models across England is a similar finding to an audit of 6MR services a decade ago [16]. Results of this survey indicate that differences in features of the 6MR appear to be associated with who the provider organisation of the service is rather than sociodemographic factors related to the local population. This suggests that the design and implementation of 6MR services may be based more on the preferences of provider organisations rather than local context. The current study helps to articulate the current level of variation within 6MR implementation and provides an indication of which factors would be beneficial to explore in future studies.

The range of service delivery models implemented is unsurprising in a complex intervention of this nature, especially where there remains a lack of clarity with regards to the ideal structure and content of the 6MR service provision. In future studies, furthering an understanding of the nature of this variation and why this variation exists will help to construct theory regarding the key components of the review, as well as the adaptable features. An analytical framework proposed by Sutherland and Levesque [21] suggest analysing variation in terms of that which is warranted and that which is unwarranted. Using this framework, relevant factors that may shape warranted variation in the context of 6MRs include the patients’ needs and the adaptation of recommendations in response to context. In the current survey, it is encouraging to see a large proportion of services offering a variety of delivery methods within their services. Doing so provides flexibility for the service users, allowing them to access the 6MR in the most convenient and appropriate way for them as individuals. Variation of this nature allows possible core components of the 6MR to be maintained whilst the adaptable periphery is adjusted to optimise the success of the intervention within a local setting [17].

Conversely, unwarranted variation of the 6MR may be shaped by the organisational design of the service, the needs and preferences of the providers, and the lack of a sufficient evidence base for 6MRs [21]. The finding that elements of the structure and processes of 6MR services are most often associated with the provider organisation may therefore lead to variation that is unwarranted. In support of this, a multiple case-study approach [22] found that the focus of the 6MR differed depending on the provider, with stroke nurse specialists providing a more medicalised model and Stroke Association representatives being more socially orientated. Whilst there are clear benefits to both models, providers need to ensure where possible that their services are designed to meet the needs of the local population and the context within which they sit, rather than the ease and preference of the organisation.

Variation was noted in the data collection tools used, with a large number of services adjusting existing tools or developing ‘in-house’ forms, despite various evidence-based tools within the literature. Whilst data collection tools can provide structure to a consultation and reduce errors [23], operationally, it is difficult to standardise the heterogenous needs of stroke survivors into a single checklist. This is demonstrated by the lower number of services that covered spasticity in the review process, potentially due to the fact that spasticity is not included in the most commonly used tool, the GM-SAT. The limitation of any single data collection tool may explain why approximately a quarter of respondents in the survey felt it necessary to use more than one. The Stroke Patient Concerns Inventory was recently developed by Chesworth and colleagues [24] to enable individualised, patient-led and tailored consultations. This method may ensure a holistic approach to the 6MR by focussing on the issues and needs most pertinent to the individual stroke survivor. However, further evaluation of this approach is required before wide-scale implementation in this setting.

Results from the survey also appear to highlight an apparent health inequality. Whilst 89% of services would carry out the 6MR in the patient’s own home, only 40% of services would do so in care homes. The reasons for this were not explored in this survey, however, stroke survivors in care homes were highlighted by a number of respondents as a group that is underserved by current 6MR services. The lack of access to healthcare services is well-documented in this group [25, 26] and, in particular, their inability to access the stroke review process [27, 28]. Providers should be aware of how the structure of their service may exacerbate this, and other, health inequalities.

Data collection to evaluate the success of services and to better understand the needs of the local population was lacking. Respondents appeared reliant on SSNAP data to evaluate their processes rather than evaluating the quality or effectiveness of the service they provide. However, evaluation of the 6MR is difficult given the lack of a clear intention or purpose. An often-cited function of the 6MR in the literature is to identify the unmet needs of stroke survivors. This was also the most commonly selected purpose by respondents in the survey. However, mere identification does not ensure that the problem can be, or will be, ameliorated [22] and there is a risk that the review depreciates to a ‘tick box exercise’ if the input ceases at this point. It is important that services are able to evaluate the impact they are having on individual stroke survivors. Clarity on the purpose and intended outcomes of the 6MR within the evidence base would help to achieve this ambition.

### Limitations

The main limitation of this study was that the sociodemographic factors explored were classified based on the subjective considerations of the respondent, rather than being data-driven. Whilst this was done to ensure anonymity of services, it may have caused a misclassification bias [29]. Therefore, caution must be applied during interpretation so as not to inflate the significance of these results. A further limitation is that it was not possible to quantify and explore all contextual factors that may have influenced the findings. Other factors such as the cultural and ethnic diversity of local populations or the infrastructure of local health and transport facilities may have enhanced understanding of the impact of context on this complex intervention.

Survey research of this type is limited by the inherent biases of this method of data collection. Firstly, there may have been a degree of self-selection bias in which those providers with a particular interest in 6MRs or with a clear service provision were more likely to respond [30]. In this respect, it was reassuring to obtain responses from a spread of providers across the country thereby limiting the effect that self-selection bias may have on the results.

Social desirability bias, in which respondents report what they feel is a more socially acceptable response rather than a reflection of true practice [31], is another consideration which may influence findings. Steps were taken within the design of the survey to limit this effect, such as randomising the order of multiple-choice questions to prevent assigning a level of importance to one answer over another. Nonetheless, the desire for services to report positively may have inflated the interpretation of current 6MR provision.

## Conclusions

There is a high degree of variation with regards to how the 6MR is undertaken in current practice. Exploration of potential factors driving this variation suggests that the provider organisation may be a factor that warrants further investigation in future studies. Service providers should be aware of this potential influence to ensure the structures and processes they have in place are optimised for the needs of stroke survivors within their localities. To support this, there is a need to ensure data is collected that better describes local populations so that services can be specifically tailored to their unique needs.

The absence of a theoretical underpinning to better articulate how, and under what circumstances, the 6MR should work may contribute to the observed variation in the current study. Further work is planned to explore this variation in more depth through a multiple case study analysis, with the hope of identifying the core components of the 6MR to enable programme theory development. Doing so would better articulate the anticipated outcomes of the 6MR so that its effectiveness can be evaluated in future studies.

## Supplementary Information

Additional file 1. Online survey. This additional file contains the finalised version of the online survey used in the study.

Additional file 2. Expanded version of Table 5. This additional file contains an expanded version of Table 5 showing further comparisons between provider organisations.

Additional file 3. Percentage of services that cover each ‘need’ during the 6MR. This additional file contains a bar chart displaying the percentage of services that cover each need during the 6MR.

Additional file 4. Percentage of services able to make onward referrals to other services. This additional file contains a bar chart displaying the percentage of 6MR services that are able to make onward referrals to other services.

## Data Availability

The datasets used and analysed during the current study are available from the corresponding author on reasonable request.

## Abbreviations

6MR: Six Month Review for Stroke Survivors
CFIR: Consolidated Framework for Implementation Research
GM-SAT: Greater Manchester Stroke Assessment Tool©
GP: General Practitioner
IQR: Interquartile Range
LUNS: Longer-term Unmet Needs after Stroke
NHS: National Health Service
PSC: Post-Stroke Checklist
SSNAP: Sentinel Stroke National Audit Programme
