# Supplementary material for: Exploring variation in the six-month review for stroke survivors: a national survey of current practice in England
Source: BMC Health Serv Res. 2025 Jan 28;25:159. doi: 10.1186/s12913-025-12323-6 (PMC11773788; doi:10.1186/s12913-025-12323-6)
Supplement: Supplementary file 1 — Additional file 1. Online survey. This additional file contains the finalised version of the online survey used in the study. [file 12913_2025_12323_MOESM1_ESM.pdf]

Introduction

Thank you for taking the time to look at this survey.

My name is Rich Holmes and I am currently doing a NIHR-funded PhD with the University of Central Lancashire. This survey forms the first stage of a project investigating the **"6-Month Stroke Review" (6MR)**. I will be exploring what we are currently doing across the country in 6-month review services, why we are doing it this way, and how we can optimise this process so that stroke survivors can access more of the long-term support they desperately need.

Please share the link to this survey with other 6MR services within your networks. We want to hear from as many 6MR services as possible so that we can build an accurate picture of what is currently provided.

It is important that you provide honest and frank answers to the questions. Please remember there are no right or wrong answers to these questions as there is no right or wrong way to provide the 6MR. We are more interested in your thoughts and opinions of your individual service. Your answers will be kept strictly confidential and not shared with your managers. There is the option to provide your email address at the end of the survey if you would like to hear about the results. Please be assured that your email will be stored separately to your responses to ensure your anonymity.

The survey will take approximately **12-15 minutes to complete.**

Please ensure you have read and understood the information sheet (available here: Participant information sheet rhv1.1) before you decide whether or not to participate.

If you have any further questions regarding the survey or the project as a whole, or if you require any additional support to complete the survey, you can contact me at richard.holmes8@nhs.net.

Thanks again for your support with this important research.

Consent

I confirm that I have read and understood the Participant Information Sheet.

- ◯ Yes
- ◯ No

I understand that my participation is voluntary and I am free to stop at any time up until I click the 'Submit' button on the last page of the survey.

- ◯ Yes
- ◯ No

I am aware that my answers will be treated confidentially and will not be linked to myself, or the service I represent, ensuring that I remain anonymous.

- ◯ Yes
- ◯ No

I agree to take part in this study.

- ◯ Yes
- ◯ No

Validation

Please can you confirm that your service provides six month reviews (6MR) for stroke survivors?

- ◯ Yes
- ◯ No

Please can you confirm that you are the service lead for the 6MR service **OR** have responsibility for the delivery of the service.

- ◯ Yes
- ◯ No

Service Demographics

What is the name of your service?

(NB. This information will only be used to ensure we avoid receiving multiple responses from

services. The name of your service will not be directly linked to your answers to the rest of the survey.)

What is your job title?

Which Integrated Stroke Delivery Network (ISDN) are you part of?

Which Integrated Care Board is your service commissioned by?

Region

Integrated Care Board

Approximately how old is your service in years?

- ☐ 0-3 years
- ☐ 4-5 years
- ☐ 6-10 years
- ☐ Over 10 years

Service Structure & Processes

Who is the provider organisation of your 6MR service?

- ○ Charitable Organisation
- ○ GP Surgery
- ○ Acute NHS Trust
- ○ Community NHS Trust
- ○ Private company
- ○ Community Interest Company (CIC)
- ○ Other (Please state)

Which of the following professionals carry out the 6MR in your service?
(Please tick all that apply)

- ☐ Charitable Sector Employee
- ☐ District/Community Nurse
- ☐ GP

- ☐ Occupational Therapist
- ☐ Physiotherapist
- ☐ Rehabilitation Assistant / Support Worker
- ☐ Rehabilitation Consultant
- ☐ Social Worker
- ☐ Specialist Nurse
- ☐ Speech and Language Therapist
- ☐ Stroke Consultant
- ☐ ____________ Other (Please state)

How is the 6MR delivered?
(Please tick all that apply)

- ☐ Post
- ☐ Face-to-face
- ☐ Telephone
- ☐ Virtual
- ☐ ____________ Other (Please state)

Where is the 6MR carried out?
(Please tick all that apply)

- ☐ Patient's home
- ☐ Clinic
- ☐ GP Surgery
- ☐ Residential/Nursing Home
- ☐ Community centre
- ☐ ____________ Other (please state)
- ☐ Not applicable (i.e. no reviews provided face-to-face)

Approximately how long does the 6MR take to complete?
(Please state time in minutes)

Direct time (i.e. time with the service user/patient) ____________

Indirect time (i.e. completing paperwork, referrals) ____________

Who receives a copy of the 6MR outcome documentation?
(Please tick all that apply)

- ☐ Service user/Patient

- ☐ GP
- ☐ Consultant/Medical records
- ☐ [ ] Other (please state)
- ☐ Not applicable (i.e. no outcome documentation is produced)

Where can DIRECT (i.e., not via a GP or other provider) referrals be made for additional post-stroke support services and rehabilitation as a result of unmet needs identified during the 6MR?
(Please tick all that apply)

- ☐ Vocational rehabilitation
- ☐ Social Worker
- ☐ Spasticity clinic
- ☐ Inpatient rehabilitation
- ☐ Audiology
- ☐ Clinical Psychology
- ☐ Driving assessments
- ☐ Dietician
- ☐ Speech and Language Therapist
- ☐ Carers Support services
- ☐ Incontinence service
- ☐ Community rehabilitation
- ☐ Pain clinic
- ☐ Outpatient rehabilitation
- ☐ Orthoptist
- ☐ Other (please state) [ ]
- ☐ Not applicable (i.e. no direct referrals - all referrals via GP)

What checklist or data collection tool is used to record data during the review process?
(Please tick all that apply)

- ☐ No tool is used
- ☐ Post-Stroke Checklist (PSC)
- ☐ Greater Manchester Stroke Assessment Tool (GM-SAT)
- ☐ Longer-term Unmet Needs after Stroke Assessment (LUNS)
- ☐ Self-devised 'in-house' forms
- ☐ [ ] Other (please state)

What items are covered during the 6MR?
(Please tick all that apply)

- ☐ Mobility
- ☐ Falls
- ☐ Medicines Management / Compliance
- ☐ Fatigue
- ☐ Secondary prevention
- ☐ Mood
- ☐ Family/Carer Needs
- ☐ Cognition
- ☐ Benefits and finances
- ☐ Continence
- ☐ Sleep pattern
- ☐ Activities of Daily Living

- [ ] Swallowing
- [ ] Spasticity
- [ ] Participation in community life/leisure
- [ ] Relationships/Sex
- [ ] Communication
- [ ] Pain
- [ ] Driving, transport and travel
- [ ] Hearing
- [ ] Other (Please state) ______
- [ ] Employment
- [ ] Vision

Population Served

Thinking about the locality you serve, would you describe the geography as mostly..........

- ○ Rural
- ○ Semi-rural
- ○ City

Thinking about the locality you serve, would you describe the level of deprivation as mostly..........

- ○ High levels of deprivation
- ○ Moderate levels of deprivation
- ○ Low levels of deprivation

In your area, are there specific groups of patients that you feel are underserved by the 6MR? *(E.g. people of certain ethnic groups, people of certain age groups, people with specific disabilities etc.)*

If so, please describe which groups and any ideas you have about why they are underserved.

______

Do you collect data on people who do not take up the 6MR?

- ○ Yes
- ○ No

What data do you collect on those that do not take up the 6MR?

______

Have you observed any themes or trends with regards to groups of patients that do not take up the 6MR?
Please describe.

What would you estimate is a typical spread of age groups within your caseload?
Please provide a percentage (%).

50 and under	0
51-65	0
66-80	0
81 and over	0
Total	0

Purpose and Outcomes

What is the purpose of the 6MR?
Please choose up to 3 answers of what you feel are the main purposes.

- [ ] Provide emotional support
- [ ] Onward referral to specialist services (i.e. spasticity/pain clinic/continence etc)
- [ ] Provide personalised care
- [ ] Identify unmet needs
- [ ] Data gathering to inform commissioning need
- [ ] Provide information / advice / signposting
- [ ] Provide treatment/intervention
- [ ] Identify carer needs
- [ ] Onward referral to rehabilitation
- [ ] Secondary prevention
- [ ] Other (please describe)

For each of the identified purposes, how well do you feel your service achieves this?

	Not well at all	Slightly well	Moderately well	Very well	Extremely well	
	0				100	
» Identify unmet needs						
» Provide information / advice / signposting						
» Secondary prevention						
» Data gathering to inform commissioning need						
» Provide personalised care						

	Not well at all	Slightly well	Moderately well	Very well	Extremely well	
	0				100	
» Onward referral to specialist services (i.e. spasticity/pain clinic/continence etc)						
» Onward referral to rehabilitation						
» Identify carer needs						
» Provide treatment/intervention						
» Provide emotional support						
» Other (please describe)						

Has the success of your 6MR service been evaluated?

If yes, please state how.

- No
- Yes - please describe

Do you use validated outcome measures?

If so, which?

- No
- Yes - please describe

Do you measure patient experience or satisfaction levels?

If so, how do you measure this?

- No
- Yes - please describe

How much do you agree with the following statements?

	Strongly disagree	Somewhat disagree	Neither agree nor disagree	Somewhat agree	Strongly agree
There is sufficient evidence that the 6MR will achieve the desired outcomes.	○	○	○	○	○
Our current method of delivering the 6MR is the best solution.	○	○	○	○	○
Our service has different pathways dependent on patient needs.	○	○	○	○	○
We are able to test new ways of working in our service if warranted.	○	○	○	○	○
Our service is easy for service users to navigate.	○	○	○	○	○
Ours is a service I am proud to be a part of.	○	○	○	○	○
Our service is value for money in relation to the outcomes we achieve.	○	○	○	○	○

How much do you agree with the following statements?

	Strongly disagree	Somewhat disagree	Neither agree nor disagree	Somewhat agree	Strongly agree
We regularly collect data on patient need.	○	○	○	○	○
The structure of our service is dictated by patient need.	○	○	○	○	○
We maintain good links with other organisations.	○	○	○	○	○
Information sharing across teams/organisations is never a problem.	○	○	○	○	○
We are more likely to adopt a new innovation if other service providers around us are already using it.	○	○	○	○	○
We regularly adapt the delivery of our service in response to SSNAP.	○	○	○	○	○
Performing well on SSNAP is a major driver in our service.	○	○	○	○	○
The structure of our service is dictated by national or local policy drivers.	○	○	○	○	○

Anything else?

Is there anything else about Six Month Stroke Reviews that we haven't asked and you think we should know?
Please feel free to leave any comments below.

Contact Details

Thank you for your time. If you would like to hear about the results of this survey please provide your email address below. (Your email address will be stored separately from your responses to maintain confidentiality.)

The next stage in this research project will be to explore a small number of 6MR services in more detail to better understand how the 6MR works. If you would be interested in your service being considered for this stage please leave your email address below.

Submit

By selecting 'Submit' you are consenting to participating in this study as described in the Participant Information Sheet. If you would like to review the Participant Information Sheet again before clicking 'Submit' it can be accessed by clicking on the following link (Participant information sheet rhv1.1).

FUNDED BY
NIHR | National Institute for Health and Care Research

Powered by Qualtrics
