# Supplementary material for: Exploring variation in the six-month review for stroke survivors: a national survey of current practice in England
Source: BMC Health Serv Res. 2025 Jan 28;25:159. doi: 10.1186/s12913-025-12323-6 (PMC11773788; doi:10.1186/s12913-025-12323-6)
Supplement: Supplementary file 2 — Additional file 2. Expanded version of Table 5. This additional file contains an expanded version of Table 5 showing further comparisons between provider organisations. [file 12913_2025_12323_MOESM2_ESM.pdf]

		Acute NHS Trusts (*n=19*)	Community NHS Trusts (*n=38*)	Charitable Organisations (*n=27*)	Others (*n=8*)
**Professionals Involved**	**Nurse**	74%	71%	0%	50%
	**Charitable Sector Employee**	0%	0%	100%	0%
	**Allied Health professional**	21%	45%	0%	13%
	**Support Worker / Assistant**	5%	39%	0%	63%
	**Consultant**	21%	0%	0%	0%
	**Psychologist**	0%	3%	0%	0%
**Age of Service (years)**	**0-3**	5%	13%	19%	0%
	**4-5**	16%	0%	11%	13%
	**6-10**	21%	24%	22%	50%
	**Over 10**	58%	63%	48%	38%
**Method of Delivery**	**Face-to-face**	74%	97%	100%	100%
	**Telephone**	95%	79%	96%	100%
	**Virtual**	5%	29%	70%	38%
	**Post**	0%	8%	0%	13%
**Location**	**Home**	58%	95%	100%	100%
	**Clinic**	47%	47%	0%	50%
	**Residential / Nursing Home**	21%	42%	44%	63%
	**Community Centre**	0%	5%	4%	0%
	**GP Surgery**	0%	0%	0%	13%
	**Not applicable (no reviews offered face to face)**	16%	3%	0%	0%
**Data Collection Tool**	**GM-SAT**	37%	34%	100%	75%
	**'In-house' forms**	53%	55%	7%	25%
	**PSC**	5%	3%	4%	0%
	**LUNS**	0%	0%	4%	0%
	**Other**	32%	34%	7%	13%
	**No tool used**	5%	3%	0%	0%
**Time to Complete (Minutes) – *Median (IQR)***	**Direct**	35 (30-60)	60 (45-60)	60 (50-90)	60 (55-67.5)
	**Indirect**	20 (10-30)	45 (30-60)	60 (45-120)	37.5 (30-75)
**Number of Different Delivery Methods – *Median (IQR)***		2 (1-2)	2 (1.75-3)	3 (2-3)	2 (2-3)

GM-SAT – Greater Manchester Stroke Assessment Tool; PSC – Post-stroke Checklist; LUNS – Longer-term Unmet Needs after Stroke; IQR – Inter Quartile Range
