# Supplementary figures and images for: Exploring variation in the six-month review for stroke survivors: a national survey of current practice in England
Source: BMC Health Serv Res. 2025 Jan 28;25:159. doi: 10.1186/s12913-025-12323-6 (PMC11773788; doi:10.1186/s12913-025-12323-6)

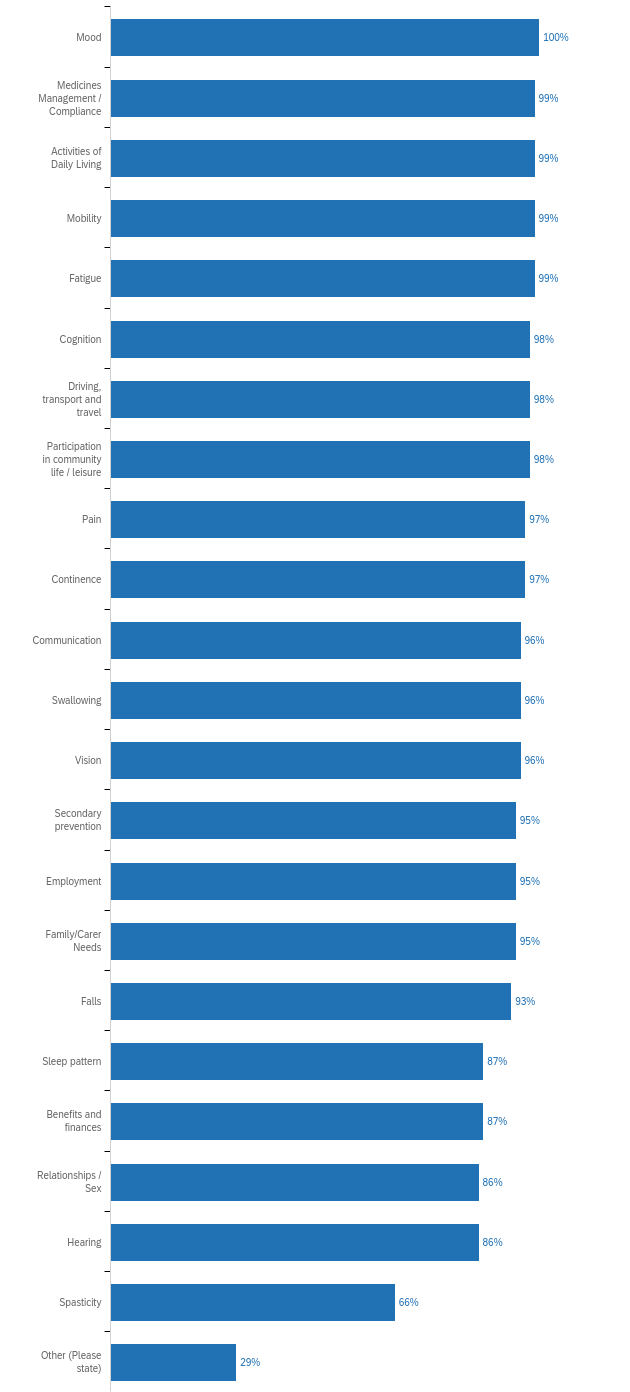

Supplement: Supplementary file 3 — Additional file 3. Percentage of services that cover each ‘need’ during the 6MR. This additional file contains a bar chart displaying the percentage of services that cover each need during the 6MR. [file 12913_2025_12323_MOESM3_ESM.png]

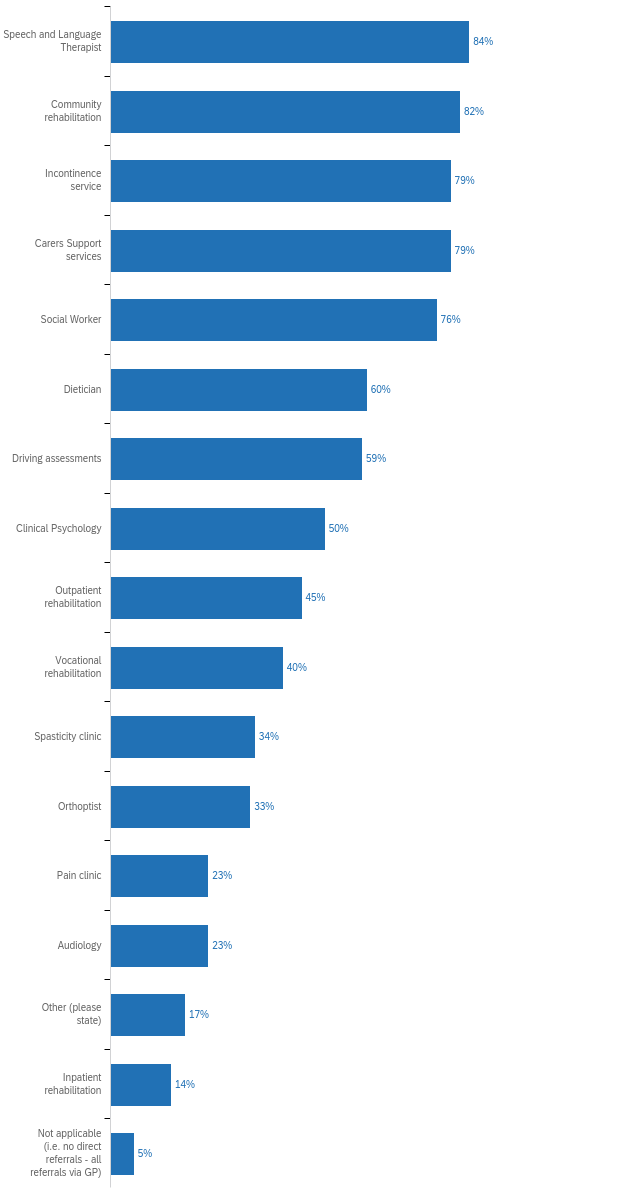

Supplement: Supplementary file 4 — Additional file 4. Percentage of services able to make onward referrals to other services. This additional file contains a bar chart displaying the percentage of 6MR services that are able to make onward referrals to other services. [file 12913_2025_12323_MOESM4_ESM.png]
